# Acute scrotal bleeding

**DOI:** 10.4103/0974-2700.70778

**Published:** 2010

**Authors:** Sudip Kumar Ghosh, Debabrata Bandyopadhyay

**Affiliations:** Department of Dermatology, Venereology, and Leprosy, R.G. Kar Medical College, 1, Khudiram Bose Sarani, Kolkata 700 004, India

**Keywords:** Angiokeratoma of fordyce, angiokeratoma of scrotum, scrotal bleeding

## Abstract

We report a case of acute scrotal hemorrhage from multiple angiokeratomas on scrotum, because of the rarity of the condition and to emphasize the importance of considering this condition in the evaluation of acute scrotal bleeding.

## INTRODUCTION

Angiokeratomas are well-circumscribed vascular lesions consisting of superficial vascular ectasia with an overlying hyperkeratotic surface.[1,2] There are many variants of angiokeratomas, namely angiokeratoma of Mibelli, solitary papular angiokeratoma, angiokeratoma circumscriptum, angiokeratoma corporis diffusum, and angiokeratoma of fordyce.[1,2] Angiokeratoma of fordyce involves the scrotum or vulva usually of the middle-aged or elderly individuals, but may also arise in the second or third decade.[2] Urethral, penile, or clitoral lesions have also been described.

## CASE HISTORY

**Figure 1:**
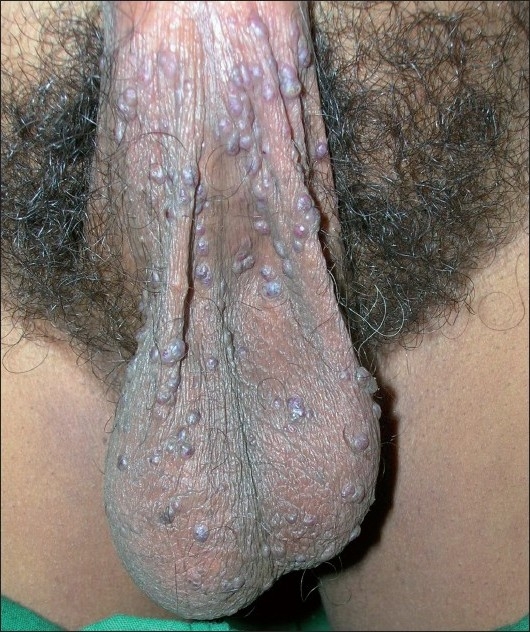
Multiple small, keratotic, vascular papules on scrotum

**Figure 2:**
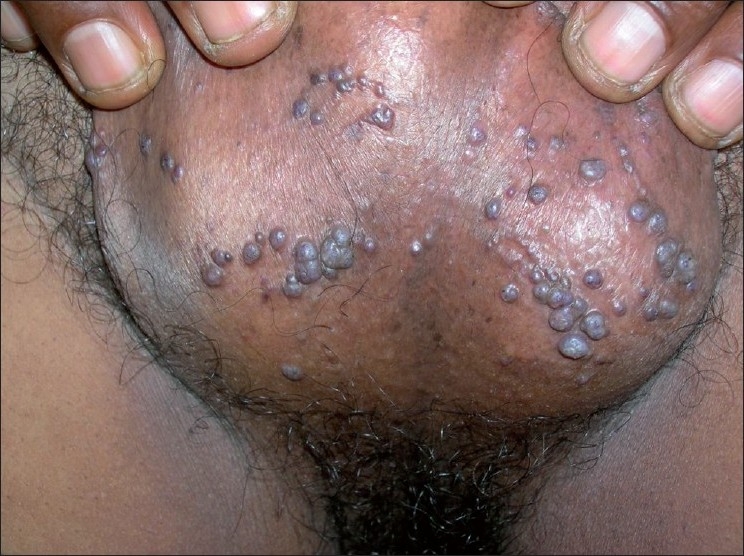
Close-up view showing multiple angiokeratoma on scrotum

A 49-year-old man presented to the emergency department with a history of profuse bleeding from his scrotum following a minor trauma. The patient had similar past episodes of recurrent scrotal bleeding spontaneously or following minor traumas over the previous 5 years. He had no history of any systemic illness. Putting firm pressure on the bleeding points stopped the bleeding. Dermatologic evaluation on the next day revealed numerous, small, bluish-red, warty, vascular nonblanchable papules on his scrotum [Figures 1 and 2]. There was no similar lesion on the other parts of his body. Systemic examination was noncontributory. Ultrasonography of the scrotum was normal. Lesional biopsy showed hyperkeratosis and marked dilatation of the dermal blood vessels. On the basis of the clinical presentation and histopathology, the diagnosis of angiokeratoma of the scrotum was made. The patient was successfully treated with electrodessication of the lesions without any further recurrence over the next 1 year of follow-up.

## DISCUSSION

John Addison fordyce first described angiokeratomas of fordyce on the scrotum of a 60-year-old man in 1896.[3] The exact incidence and pathogenesis of angiokeratoma of fordyce are unknown, but the lesions are considered common, especially in the older age group. There is a male preponderance of the disease.[4] The lesions are characterized by multiple keratotic, dome-shaped, vascular papules, 1–6 mm in diameter, that bleed spontaneously, on minor trauma or during intercourse.[2,3] Patients may even seek medical attention to rule out a sexually transmitted disease or malignancy.[3] The scrotal lesions may also be a part of the generalized angiokeratomas that occur in Fabry’s disease. The other potential precipitants are intra-abdominal masses, urinary tract tumors, and varicoceles.[4] Vulvar lesions may be associated with vulvar varicosities, oral contraceptive pills, hemorrhoids, hysterectomy, or increased venous pressure during pregnancy.[2] Histopathology of angiokeratomas shows marked dilatations of superficial dermal blood vessels with an acanthotic, hyperkeratotic epidermis. Elongated epidermis may enclose vascular channels, and a collarette may be present at the periphery of the lesions.[2]

The major reasons for morbidity of this disease are bleeding, anxiety, and overtreatment due to misdiagnosis by the treating physicians. Spontaneous resolution does not occur, and the angiokeratomas persist unless treated.[3] The patient must be reassured regarding the benign nature of the disease. The preliminary management of bleeding from angiokeratoma is to put direct pressure.[4] The specific treatment options for angiokeratoma of scrotum include electrofulguration, cryotherapy, and laser ablation, or shave excision.[1–4] The lesions may however recur after treatment, whatever may be the treatment modalities.

## CONCLUSION

In the emergency department, angiokeratoma of fordyce is an important differential diagnosis of scrotal bleeding and must be differentiated from other vascular lesions.

If the diagnosis of the disease remains uncertain, then referral to a dermatology clinic for opinion and skin biopsy is warranted.[4]
